# Supplementary material for: Pegasus, a small extracellular peptide enhancing short-range diffusion of Wingless
Source: Nat Commun. 2021 Sep 27;12:5660. doi: 10.1038/s41467-021-25785-z (PMC8476528; doi:10.1038/s41467-021-25785-z)
Supplement: Supplementary file 1 — Supplementary Information [file 41467_2021_25785_MOESM1_ESM.pdf]

Supplementary material

Fig S1 – S9

Tables S1-S3

References

Movies S1-S2

Source Data

Supplementary Figures:

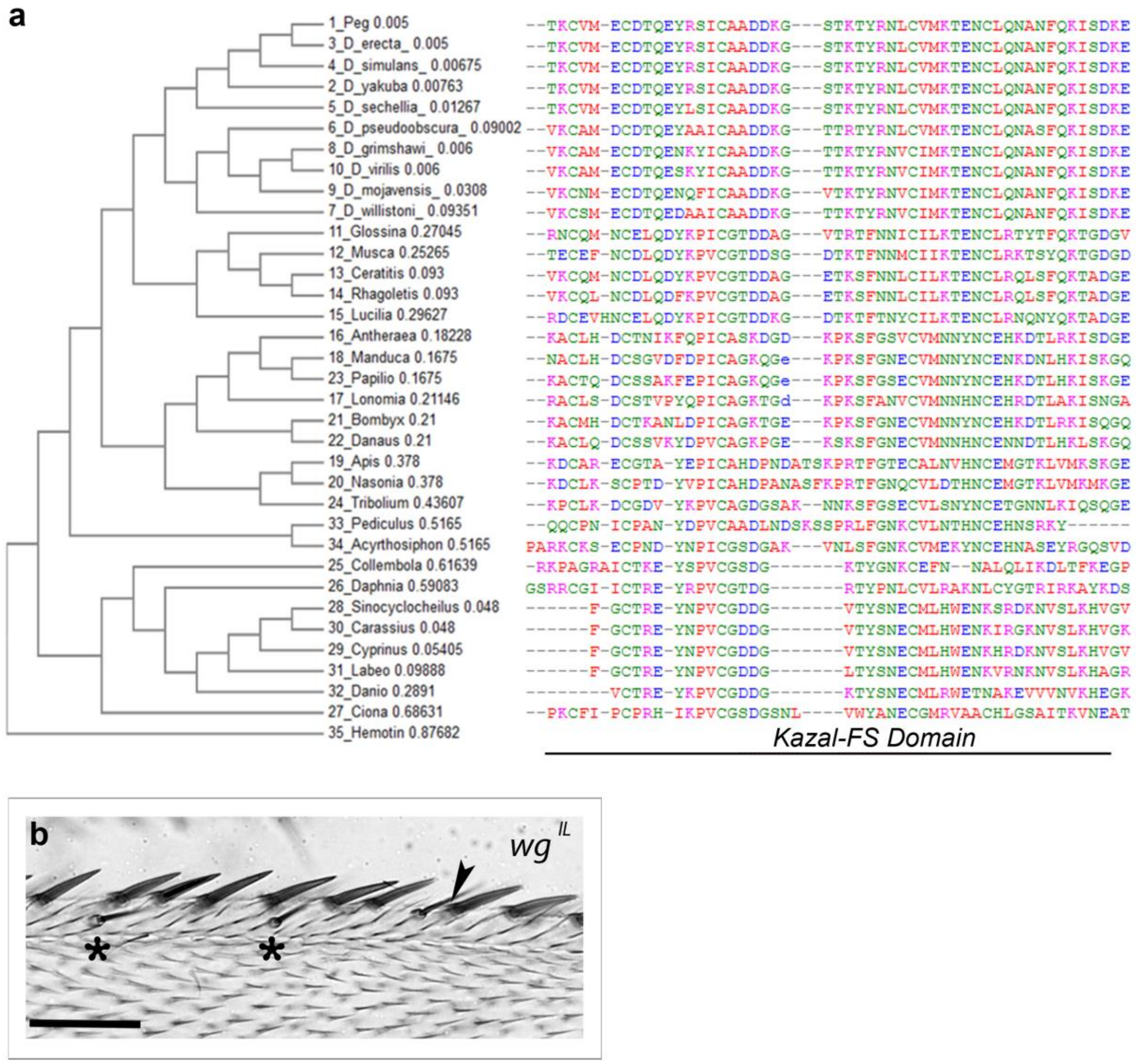

Figure S1

Figure S1 a. **Extended Phylogenetic tree and alignments of peg-like homologues in species ranging from flies to fishes,** identified by a similar homology search as in [1] . The unrelated 88aa small ORF membrane peptide Hemotin[2] was used as an outgroup for the generation of the phylogenetic tree. **b.** The wg^{IL} temperature-sensitive allele (raised at 17°C), which affects Wg secretion [3,4] generates a hypomorphic phenotype (a reduction in chemosensory bristles) similar to both the membrane-tethered Wg (Wg-NRT) allele and peg^{-} mutants (see Fig. 2a,b and Fig. 5b). Scale bar: 50µm.

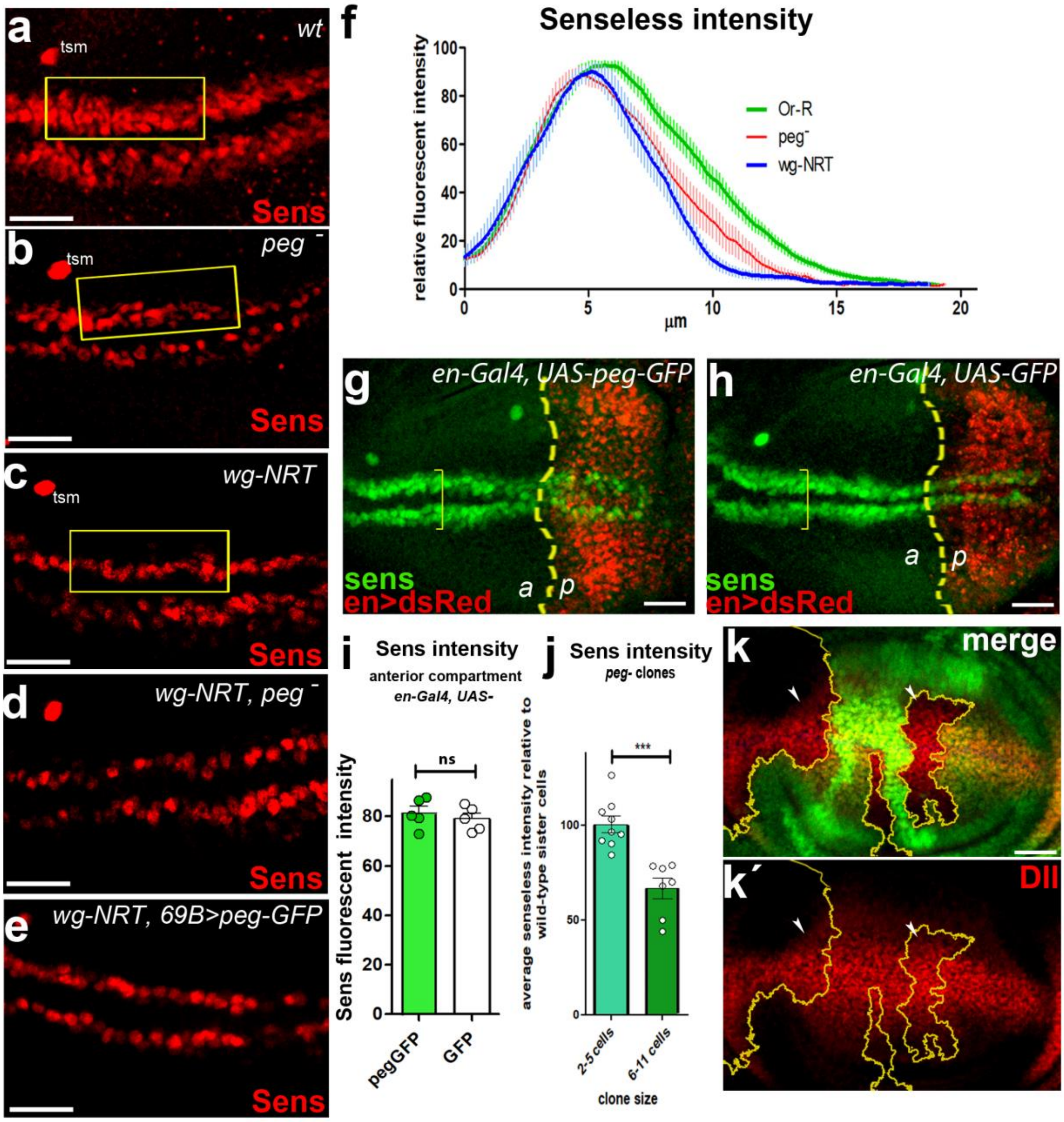
a
wt
tsm
Sens
b
peg -
tsm
Sens
c
wg-NRT
tsm
Sens
d
wg-NRT, peg -
Sens
e
wg-NRT, 69B>peg-GFP
Sens
f
Senseless intensity
relative fluorescent intensity
µm
Or-R
peg-
wg-NRT
g
en-Gal4, UAS-peg-GFP
sens
en>dsRed
a
p
h
en-Gal4, UAS-GFP
sens
en>dsRed
a
p
i
Sens intensity
anterior compartment
en-Gal4, UAS-
ns
Sens fluorescent intensity
pegGFP
GFP
j
Sens intensity
peg- clones
***
average senseless intensity relative to wild-type sister cells
2-5 cells
6-11 cells
clone size
k
merge
k´
Dll

Figure S2

Figure S2

a-c. The expression of *senseless* is significantly reduced in *peg*- mutants and in WgNRT transgenic flies compared to wt. **d-e.** *peg* removal (d) or over-expression of Peg (e) do not affect Sens expression in a WgNRT background, compare with (c). *tsm*, twin campaniform sensilla. **f.** Quantification showing the average Sens signal intensity from panels a-c relative to the maximum values. Senseless was quantified in a region of interest, represented by yellow squares in panels a-c. N=8 wing discs per genotype. Error bars represent SEM (standard error to the mean). 0 µm represents the centre of the *wg*-expressing stripe (black stripe between Sens red stripes). Error bars represent SEM. **g-h.** Wider view of panels d-e in Figure 4, showing that over-expression of PegGFP with *en-Gal4* produces a posterior compartment (p) increase in *sens* expression (g), compared to the control posterior compartment expressing GFP-only (h), and has no effect on the anterior compartment (*a*); quantified in (i). DsRed (red) labels PegGFP expressing cells. Note native Sens expression is naturally lower in the posterior (*p*) v.s. anterior (*a*) compartment. **i.** Quantification of Sens fluorescent intensity in the anterior compartment of imaginal discs from panels g-h. showing no significant difference in Sens fluorescent intensity between PegGFP and GFP controls (one-tailed t-test, N=5 individual imaginal discs per genotype, P=0.27), Error bars represent SEM **j**. Quantification of the effect of *peg* mutant clones near the wing margin on Sens expression according to their size. Small clones (<6 cells in width) have no effect on expression whereas larger ones (6 cells or more) produce a significant reduction of Sens (one-tailed t-test, N=17 individual clones, P=0.0001). Error bars represent SEM. **k.** Large *peg*$^{-}$ clones (> 5 cells in width) identified by loss of GFP (green, arrowheads) show no effect on Dll expression. Source data are provided as a Source Data file. Scale bars: a-c, g-h, k: 20µm.

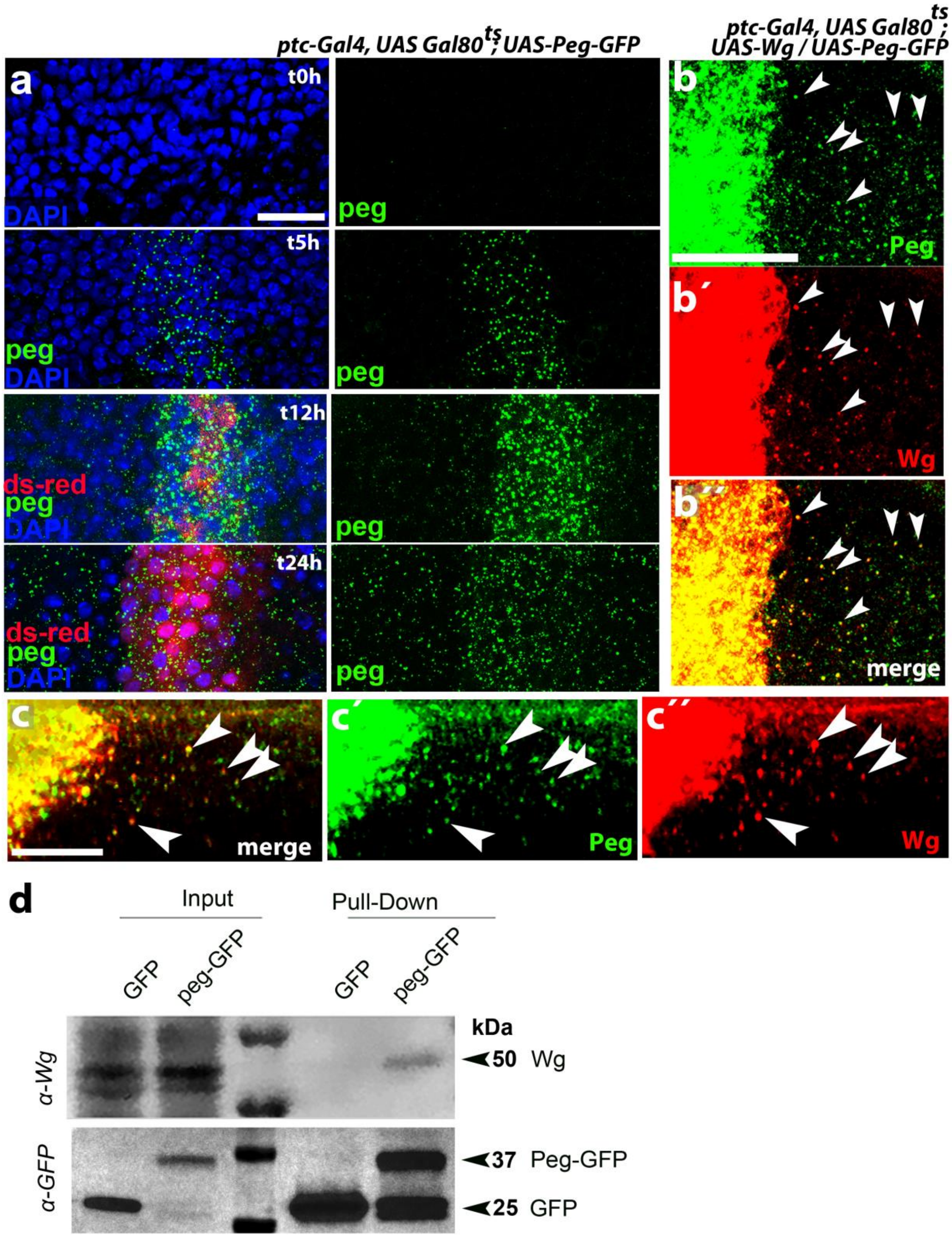
ptc-Gal4, UAS Gal80ts; UAS-Peg-GFP
ptc-Gal4, UAS Gal80ts; UAS-Wg / UAS-Peg-GFP
a
t0h
DAPI
peg
t5h
peg
DAPI
peg
t12h
ds-red
peg
DAPI
peg
t24h
ds-red
peg
DAPI
peg
b
Peg
b´
Wg
b´´
merge
c
merge
c´
Peg
c´´
Wg
d
Input
Pull-Down
GFP
peg-GFP
GFP
peg-GFP
α-Wg
α-GFP
kDa
50 Wg
37 Peg-GFP
25 GFP

Figure S3

Figure S3 a. Time-line of induction of PegGFP expression with *ptcGal4-UASGal80*^{ts} by temperature shift (18°C to 29°C) prior to dissection shows that PegGFP is secreted. The time of induction prior to dissection is indicated in each panel. DsRed (red) labels expressing cells, whereas GFP (green dots) reveals the spread of the Peg peptides, note that DsRed has a delay in expression compared to GFP, and cannot be detected after 5h of induction. **b.** Secreted co-localisation of UAS-PegGFP and UAS-Wg (arrowheads) co-expressed for 12h as in (a). **c.** Orthogonal Z-axis view of (b), showing co-localisation between PegGFP and Wg (arrowheads) along the apico-basal axis. **d.** Pull-down with anti-GFP from larvae expressing PegGFP ubiquitously driven by *da-Gal4* yields a 50–kDa Wg-specific band. GFP-only negative controls show no signal despite similar protein inputs. Source data are provided as a Source Data file. Scale bars: a-b: 20µm; c: 10µm

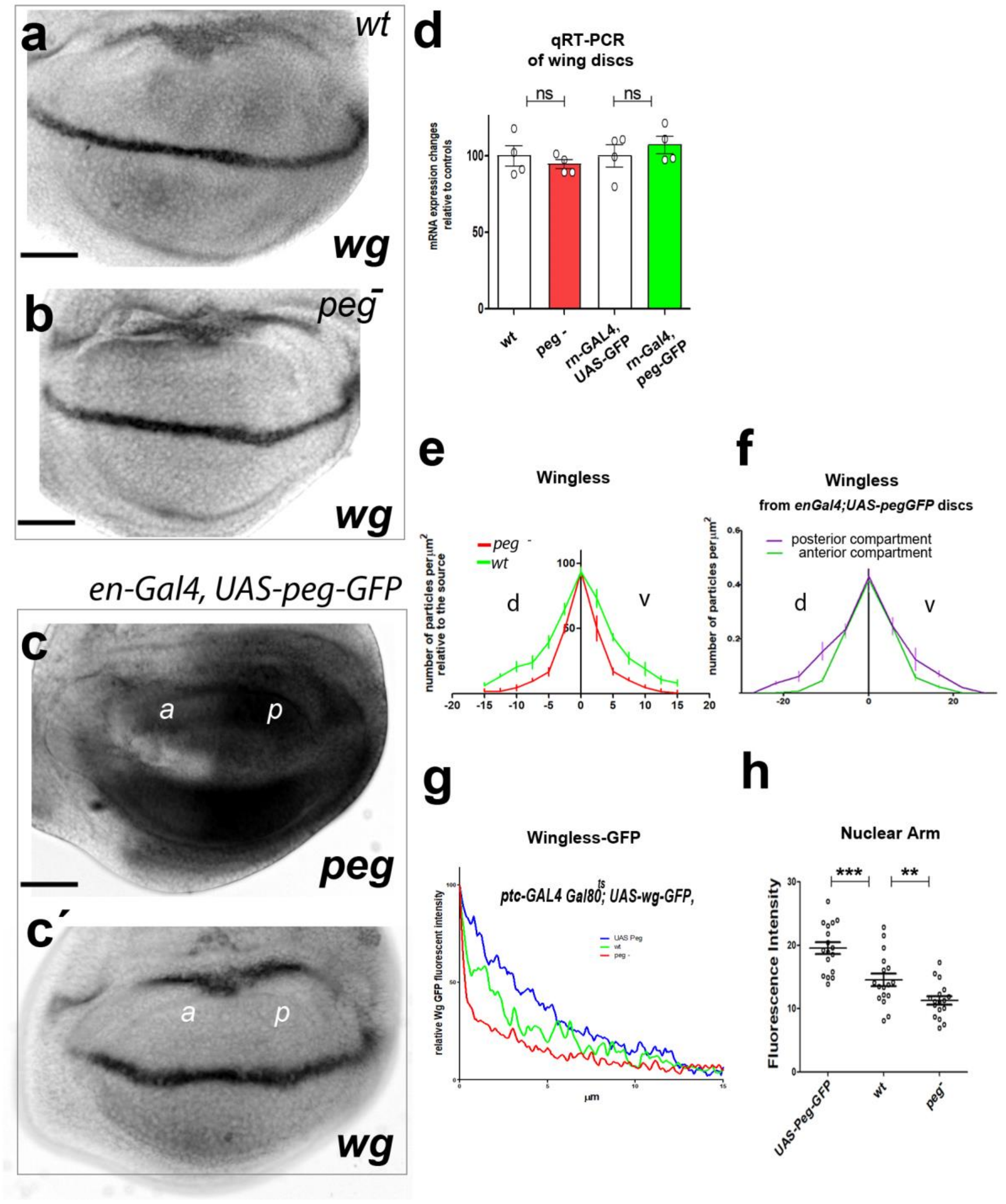

Figure S4

Figure S4. a-b. *In-situ* hybridization showing that *peg* null mutants (b) present no changes in Wg mRNA expression compared to wt (a). **c.** *In-situ* hybridization showing *wg* (c´) and *peg* (c) mRNA expression when *pegGFP* is over-expressed in the posterior compartment (*p*) with *en-Gal4*. *peg* over-expression is noticeable as the probe hybridizes both endogenous *peg* (See Fig.

1c´) and transgenic *pegGFP* mRNAs, yet it has no effect on *wg* mRNA expression. *a* and *p* indicate anterior and posterior compartments. **d**. Quantification of four biological replicas of *wg* mRNA levels by qPCR. The values were obtained using the ΔΔQC values of the *wg* amplicon against the *rp49* control and the experimental conditions against the controls. All the values were then normalized to controls. Note that there are no significant *wg* mRNA expression changes between wing imaginal discs mutant for *peg* and wt discs, nor between discs over-expressing Peg and controls (one-tailed t-test, from 4 biological replicates, P=0.24 and 0.12, respectively). Error bars represent SEM. **e.** Quantification from panels a-b in Figure 4. Average number of fluorescent Wg particles per μm^2 on either side of the presumptive wing margin (dorso/ventral (d/v) boundary), normalised to its centre (line at 0 µm). Error bars represent SEM (n=4 individual profiles per genotype). **f.** Quantification from panels c in Figure 4. Number of fluorescent Wg particles per μm^2 on either side of the presumptive wing margin (dorso/ventral (d/v) boundary, line at 0 µm) comparing the anterior (purple) vs posterior (green) compartment, in imaginal discs over-expressing PegGFP with *en-Gal4.*Error bars represent SEM, (n=4 individual profiles per genotype). **g.** Average profiles of WgGFP intensity, quantified from the regions of interest (yellow rectangles) shown in Fig.4 g-i. adjacent to the *ptc* domain cells driving the expression of WgGFP. The values are relative to maximum intensities, 0 µm represents the boundary of Wg expressing cells (n=4 individual profiles per genotype). **h.** Quantification of the nuclear Armadillo signal from Fig.4j-l showing significantly higher levels of nuclear Armadillo in wing discs over-expressing UAS-Peg, and lower levels in *peg*$^-$ wing discs compared to wing discs with a wt *peg* background (one-tailed t-test, N=17 cells, P=0.0004 and P=0.0058, respectively). Error bars represent SEM. Source data are provided as a Source Data file. Scale bars: a-c: 50 µm.

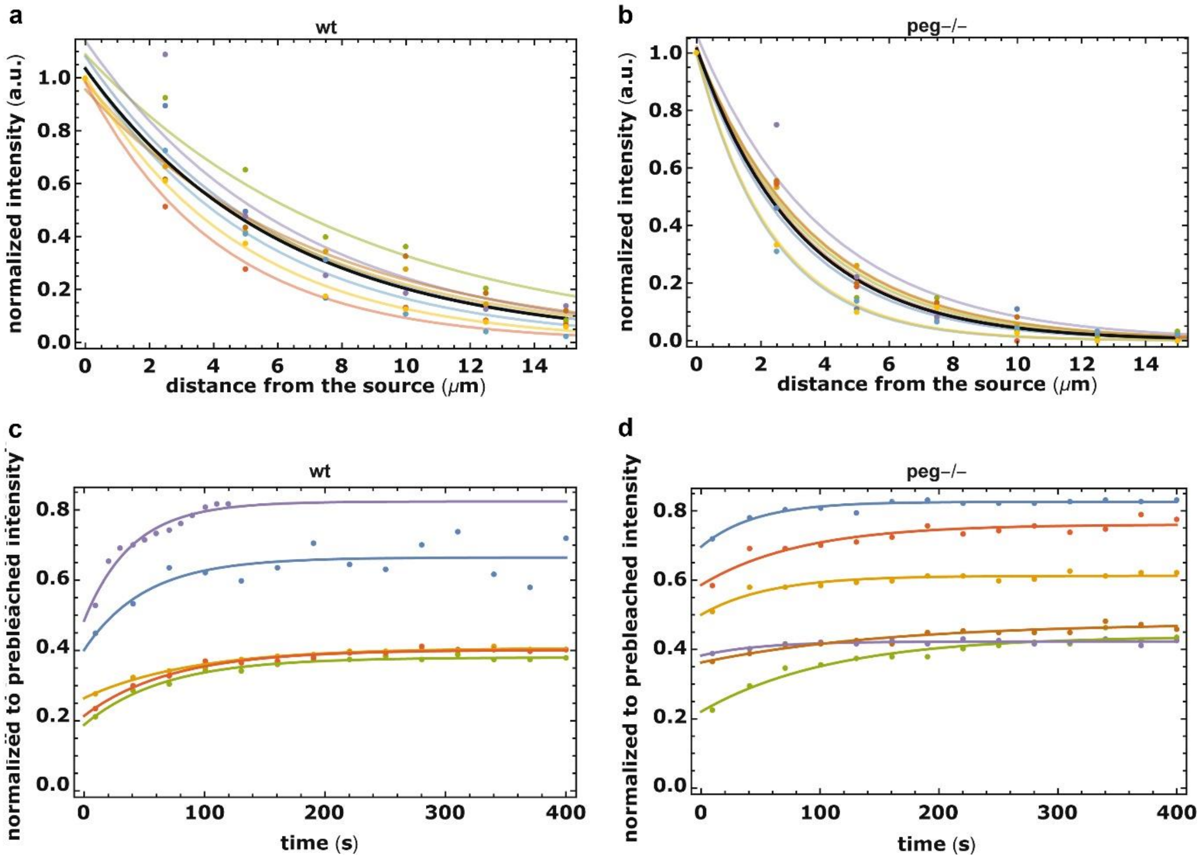

Figure S5

Figure S5

Data fitting to transport model. **a, b.** Fit of the Wg graded concentration profiles for (a) *wt* and (b) *peg*$^{-}$ to Eq.2 (fitted profiles shown as solid lines). Data (dots) is normalized to the intensity value right next to the source (position 0 in distance from the source). **c, d.** Fit of the Wg FRAP dynamics for (c) *wt* and (d) *peg*$^{-}$ (fitted curves shown as solid lines). Data (dots) are normalized to the pre-bleached intensity.

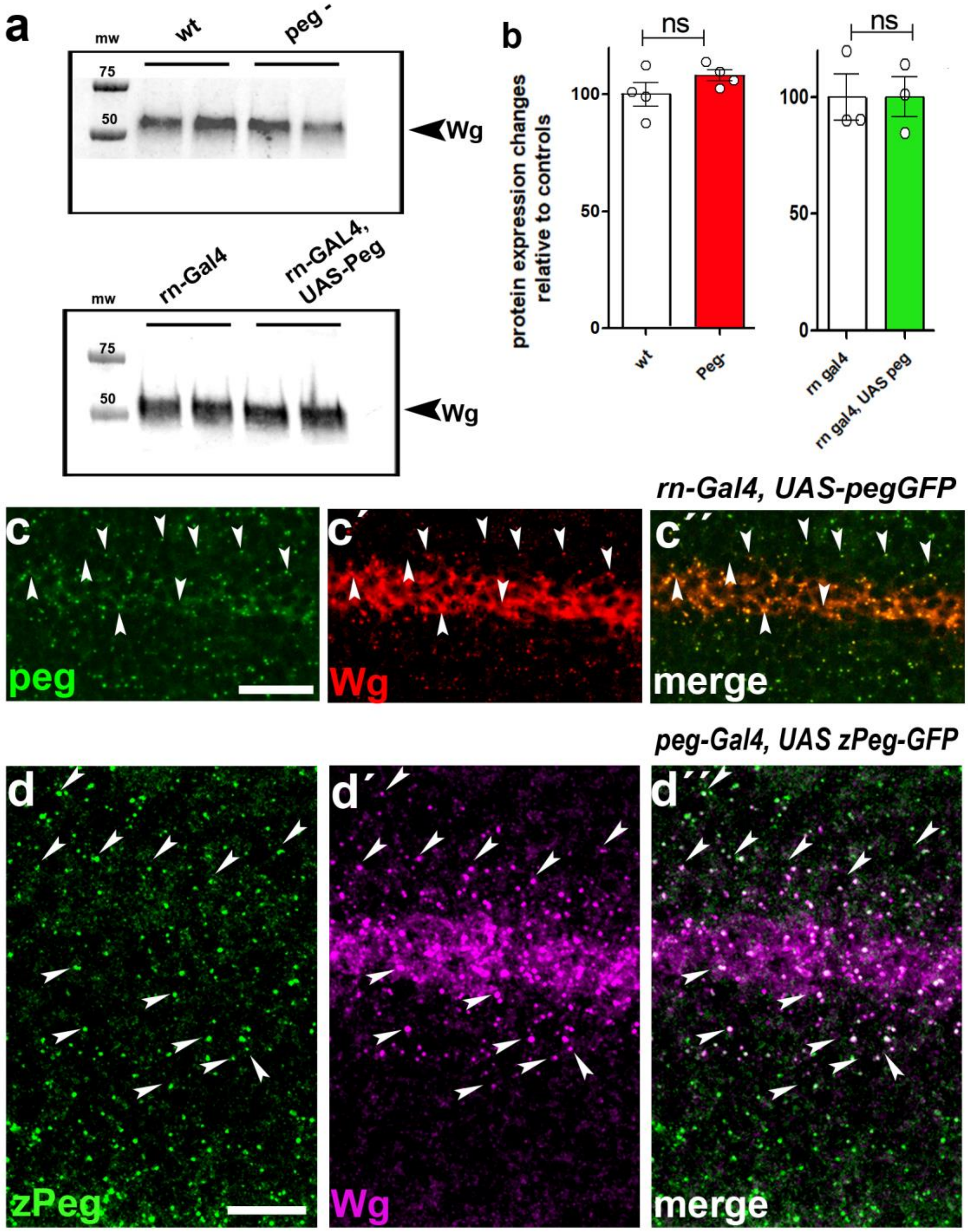
a
mw
wt
peg -
75
50
Wg
rn-Gal4
rn-GAL4, UAS-Peg
b
ns
protein expression changes relative to controls
100
50
0
wt
Peg-
rn gal4
rn gal4, UAS peg
rn-Gal4, UAS-pegGFP
c
c´
c´´
peg
Wg
merge
peg-Gal4, UAS zPeg-GFP
d
d´
d´´
zPeg
Wg
merge

Figure S6

Figure S6

a. Western Blots, from wing imaginal disc pouch extracts showing Wg protein levels, revealed with the anti-Wg antibody 4D4, in *peg*$^{-}$ mutants compared to wild-type, or in wing pouches over expressing Peg with *rnGal4*, compared to controls carrying *rnGAL4* only; quantified in (b). Wg levels remain unchanged in either *peg*$^{-}$ or Peg over-expressing imaginal discs, compared to controls. (mw: molecular weight) **b.** Quantification of three biological replicas of western blot data from imaginal discs as in (a), show no significant Wg Protein expression changes between wing imaginal discs mutant for *peg* and wt discs, nor between discs over-expressing Peg and controls (one-tailed t-test, n= 3 biological replicates, P=0.24 and 0.36 respectively). Error bars represent SEM. The Wg protein levels were adjusted against total protein loads, and normalized against the controls (see methods). **c.** *rnGal4* driven PegGFP is expressed in the wing pouch, and colocalizes with endogenous Wg, revealed with the anti-Wg antibody 4D4 (arrowheads). **d.** Danio rerio Peg homologue **z**PegGFP (green), expressed with *peg-Gal4*, co-localizes with Wg (magenta) in the developing wing margin cells (white dots, arrowheads). Scale bars: c: 15 µm ; d: 10 µm.

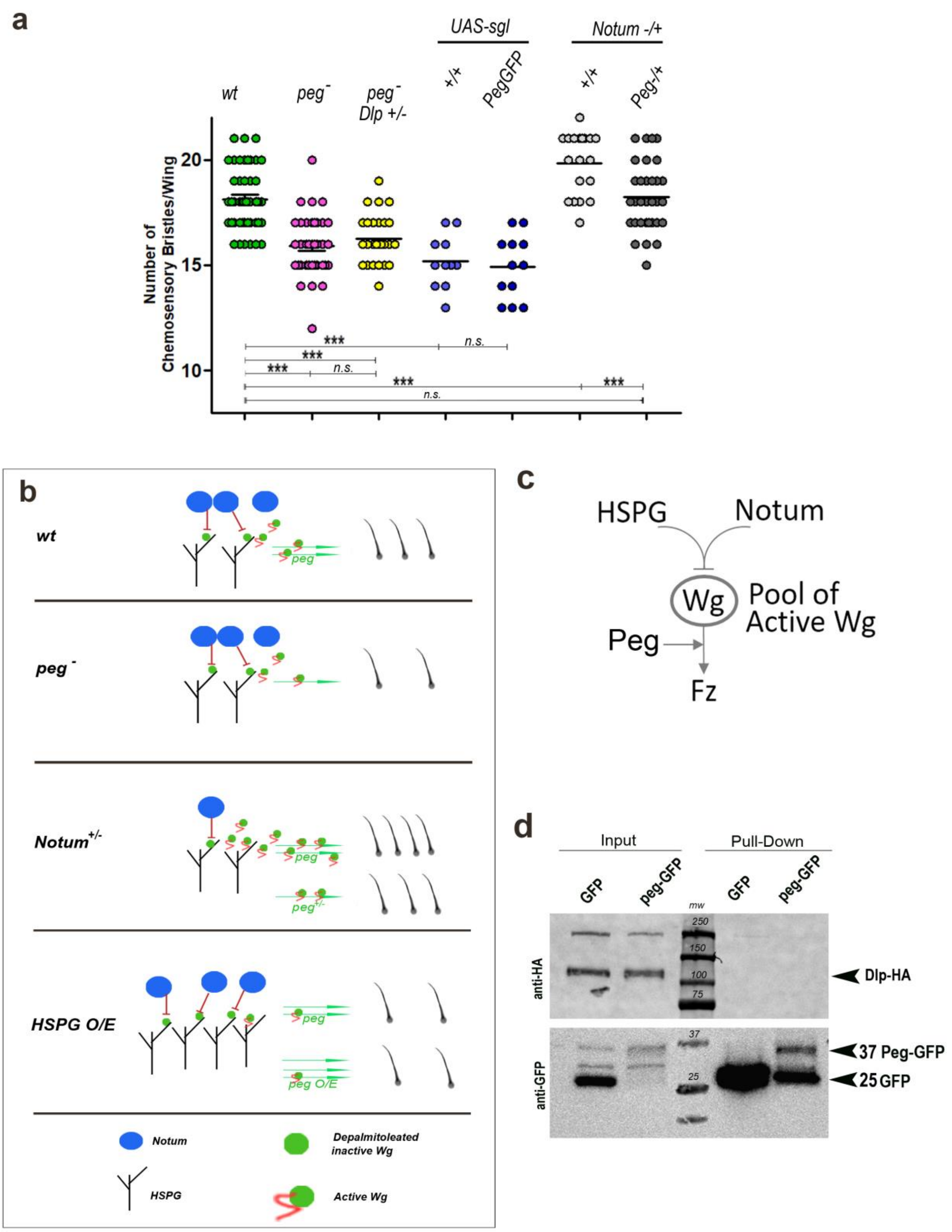
a
UAS-sgl
Notum -/+
wt
peg-
peg-
Dlp +/-
+/+
PegGFP
+/+
Peg-/+
Number of Chemosensory Bristles/Wing
20
15
10
***
n.s.
***
***
n.s.
***
***
n.s.
b
wt
peg
peg-
Notum+/-
peg
peg+/-
HSPG O/E
peg
peg O/E
Notum
Depalmitoleated inactive Wg
HSPG
Active Wg
c
HSPG
Notum
Wg
Pool of Active Wg
Peg
Fz
d
Input
Pull-Down
GFP
peg-GFP
mw
GFP
peg-GFP
anti-HA
250
150
100
75
Dlp-HA
anti-GFP
37
25
37 Peg-GFP
25 GFP

Figure S7

Figure S7 Peg acts downstream HSPGs. a. Quantification of chemosensory bristles in different genetic backgrounds. *peg* mutants, show a significant reduction compared to wild-type. Over-expression of *sugarless (sgl),* an enzyme required for HSPG synthesis, leads to loss of chemosensory bristles, and over-expression of Peg (*rn-Gal4, UAS-sgl / UAS-pegGFP*) does not rescue this phenotype. Reduction of Notum (in a null heterozygous background, *w; $Notum^{I6}$ /+),* a carboxylesterase which in cooperation with HSPGs removes an essential palmitoleate moiety from Wg, inactivating it, produces an excess of chemosensory bristles. This phenotype is rescued by reduction of *peg,* in a double heterozygous background (*w; $Notum^{I6}$ /peg^{Del1}*), suggesting that, optimal transport of Wg with Peg is required for this ectopic bristle phenotype to manifest. Reduction of Dlp in a *peg* mutant background (*dlp^{MH20}/+; peg-/-)* has no effect on the *peg* mutant chemosensory britstle phenotype. (***: $p<0.001$, **:$p<0.01$) assessed by one-tailed t-test, see Table S1 for statistical analyses. Error bars represent SEM. **b.** Schematic of the effects of Notum, HSPGs, and Peg, based on our results, suggesting that the levels of HSPGs/Notum would determine the pool of active Wg that Peg can subsequently act upon. In wild type flies, Peg is required to achieve optimal transport levels of active Wg and activate short-range proneural target genes. Removal of Peg affects the transport of active Wg, leading to a reduction of proneural gene expression and hence chemosensory bristles. Reduction of Notum increases the pool of active Wg, which in the presence of wt Peg levels leads to an increase in chemosensory bristles, but not if Peg levels are reduced. Increasing the levels of HSPGs by over-expressing Sgl leads to a reduction of chemosensory bristles, most likely by increasing the cooperation between HSPGs and Notum and reducing the pool of active Wg [19]. Over-expression of Peg (enhancing Wg transport) cannot compensate for this reduced Wg pool. **c.** Notum acts together with HSPGs to inhibit Wg by de-palmitoleation. The remaining Wg constitutes a pool of active Wg, which requires the transport enhancing function of Peg for proper short-range activation

of pro-neural genes and patterning of the wing margin. **d.** Pull-down with anti-GFP from larvae expressing PegGFP and dlp-HA in imaginal discs using *rn-Gal4 UAS-pegGF*P, showed no signal in the pull down fraction when revealed with anti-HA (like the UAS-GFP-only negative controls), despite clear signal appearing in the input fractions. Source data are provided as a Source Data file. (mw: molecular weight).

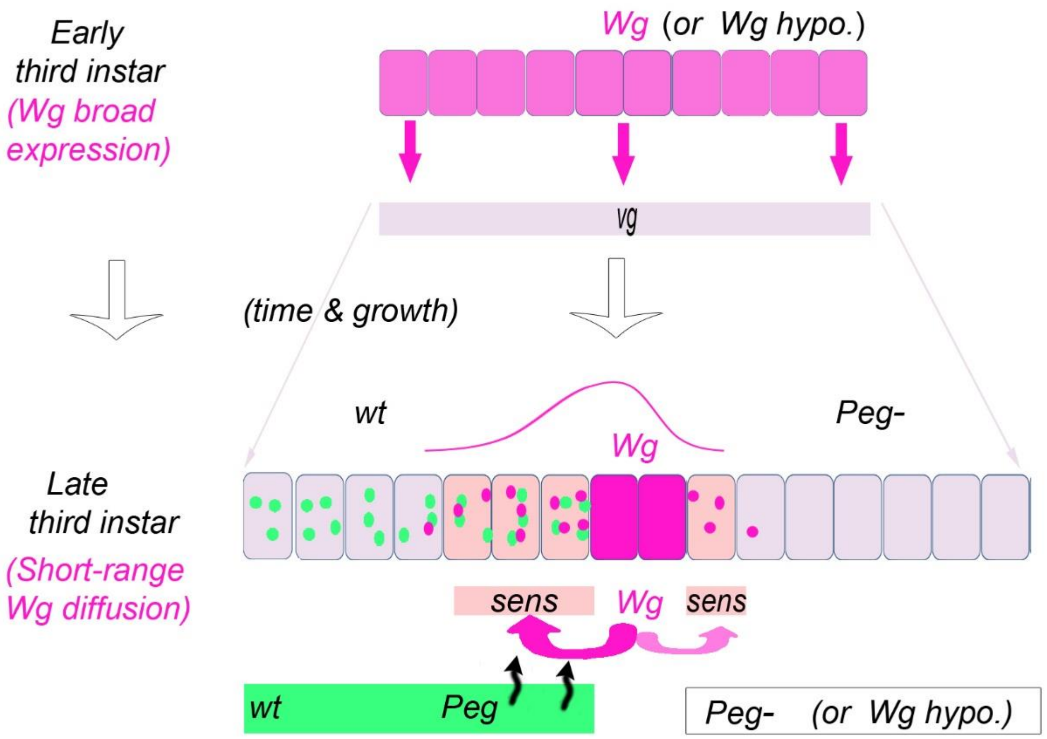

Figure S8

Figure S8

Model of *wg* and *peg* function during the development of the fly wing. During Early-mid third instar (aprox. 84 to 96 hours after egg laying, AEL), Wg protein is expressed in a developing pattern that transitorily reaches the entire developing wing and maintains the expression of the *vestigial* gene (*vg*), which is required for the growth of the wing cells [5-10]. This function does not rely on Wg diffusion and can be provided by hypomorphic, diffusion-deficient Wg (Wg hypo.) proteins such as the membrane-tethered Wg^{NRT} or Wg^{IL} at 17ºC. Later, from 96 to 132h AEL, *wg* is expressed only at the presumptive wing margin whereas Peg is expressed in the rest of the wing cells, and both are required for wing margin development [4,11] (this work). Wg activates the expression of proneural genes such as *sens* and

achaete at both sides of the *wg*-expressing cells, with an effective range of signalling of 3-4 cells. Amongst the proneural field, individual cells are selected by a Notch-mediated lateral inhibition process to become the precursors of the wing margin bristles, such as first (by about 120 AEL) the chemoreceptors, that inform the fly of its surroundings, are specified and later the mechanoreceptors that allow the fly to keep steady constant flight. Complete removal of Wg function from 96 hours AEL eliminates the entire wing margin whereas later and/or partial removal of Wg reduces the number of chemoreceptors, and then of mechanoreceptors [4,11,12]. Secreted Peg and Wg interact physically and increase Wg effective short-range diffusion to 3-4 cells. Loss of Peg reduces this range to 1-2 cells and produces a mild loss of Wg function phenotype, whereas Wg^{NRT} [13] reduces signalling even further (to 1 cell) and produces a stronger, but still not total, loss of Wg function phenotype (Figs. 2 and 5). The effective range of Wg diffusion during wing margin development is indicated by the tsm twin sensilla, which is located some 6-8 cells from the *wg*-expressing cells but is not dependant on Wg function (Figs. 1,3,4,S2; see also[12]).

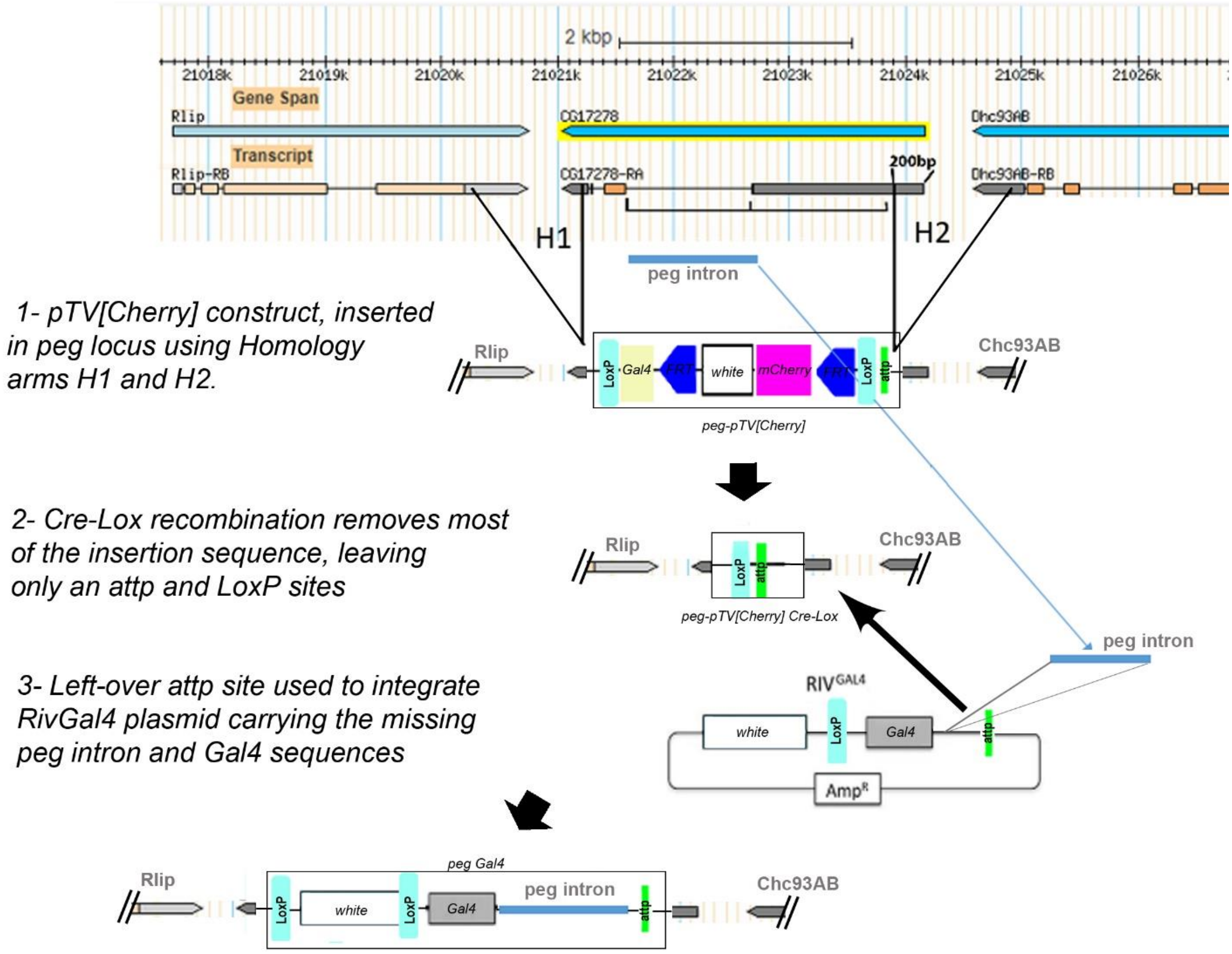

Figure S9

Figure S9. Diagram representing the changes in the *peg* genomic region after each of the steps to generate *peg*^{Gal4} by CRISPR-mediated homologous gene-editing, as described in materials and methods.

	Genotype (a vs b)	Statistical test	P	mean +/- SEM (a)	mean +/- SEM (b)	N (a)	N (b)
Fig 2e	*wt vs peg-/-*	one tailed, unpaired t test	0.0001	18.13 ± 0.2097	15.92 ± 0.2436	48	36
	wt vs pegGal4/-	one tailed, unpaired t test	0.0001	18.13 ± 0.2097	16.00 ± 0.4183	48	16
	peg-/- vs peg -/-; da>Peg-GFP	one tailed, unpaired t test	0.0001	15.92 ± 0.2436	17.69 ± 0.1999	36	35
	peg-/- vs peg -/-; da-Gal4	one tailed, unpaired t test	ns, 0.1983	15.92 ± 0.2436	16.19 ± 0.1953	36	64
	pegGal4/- vs peg -/-; da>Peg-GFP	one tailed, unpaired t test	0.0001	16.00 ± 0.4183	17.69 ± 0.1999	16	35
	pegGal4/- vs peg -/-; peg>Peg-GFP	one tailed, unpaired t test	0.0013	16.00 ± 0.4184	17.50 ± 0.1826	16	16
	pegGal4/- vs peg -/-; peg>zPeg-GFP	one tailed, unpaired t test	0.0095	16.00 ± 0.4184	17.19 ± 0.2267	16	27
	wt vs wg-NRT	one tailed, unpaired t test	0.0001	18.13 ± 0.2097	12.32 ± 0.3807	48	31
	wt vs wg-NRT; peg-/-	one tailed, unpaired t test	0.0001	18.13 ± 0.2097	12.43 ± 0.5415	48	21
	wt vs wg-NRT; peg-GFP	one tailed, unpaired t test	0.0001	18.13 ± 0.2097	13.14 ± 0.3921	48	21
	wg-NRT vs wg-NRT; peg-/-	one tailed, unpaired t test	ns , 0.4348	12.32 ± 0.3807	12.43 ± 0.5415	31	21
	wg-NRT vs wg-NRT; peg-GFP	one tailed, unpaired t test	ns, 0.0762	12.32 ± 0.3807	13.14 ± 0.3921	31	21
	wg-NRT vs wgIL	one tailed, unpaired t test	ns, 0.3695	12.32 ± 0.3807	12.48 ± 0.2245	31	25
Fig S7	*wt vs rn-Gal4, UAS sgl, UAS GFP*	one tailed, unpaired t test	0.0001	18.13 ± 0.2097	15.18 ± 0.3770	48	11
	rn-Gal4, UAS sgl, UAS pegGFP vs rn-Gal4, UAS sgl, UAS GFP	one tailed, unpaired t test	ns, 3261	14.92 ± 0.4345	15.18 ± 0.3770	12	11
	wt vs Notum/+	one tailed, unpaired t test	0.0001	18.13 ± 0.2097	19.84 ± 0.3356	48	19
	Notum/Peg- vs Notum/+	one tailed, unpaired t test	0.0004	18.22 ± 0.2866	19.84 ± 0.3356	32	19

Table S1

Table S1. Statistical analyses of the data presented in Figures 2E and S7. N: number of individual wings counted.

	Peg	*D.erecta*	*D.simulans*	*D.yakuba*	*D.sechellia*	*D.pseudoobscura*	*D.grimshawi*	*D.virilis*	*D.mojavensis*	*D.willistoni*	*Glossina*	*Musca*	*Ceratitis*	*Rhagoletis*	*Lucilia*	*Antheraea*	*Manduca*	*Papilio*
Peg	100	98.75	97.5	98.75	96.25	78.75	80	80	80	77.5	42.5	44.3	48.75	48.75	49.35	28.75	32.5	27.5
D.erecta	98.75	100	98.75	97.5	97.5	78.75	80	80	80	77.5	42.5	45.57	48.75	48.75	50.65	28.75	32.5	27.5
D.simulans	97.5	98.75	100	96.25	98.75	78.75	80	80	80	78.75	42.5	46.84	48.75	48.75	51.95	30	32.5	27.5
D.yakuba	98.75	97.5	96.25	100	95	77.5	78.75	78.75	78.75	78.75	42.5	44.3	47.5	50	50.65	28.75	32.5	27.5
D.sechellia	96.25	97.5	98.75	95	100	78.75	80	80	80	78.75	42.5	46.84	48.75	48.75	51.95	30	32.5	27.5
D.pseudoobscura	78.75	78.75	78.75	77.5	78.75	100	86.25	86.25	83.75	86.25	42.5	44.3	53.75	52.5	46.75	27.5	31.25	28.75
D.grimshawi	80	80	80	78.75	80	86.25	100	98.75	95	88.75	42.5	44.3	53.75	53.75	50.65	28.75	31.25	28.75
D.virilis	80	80	80	78.75	80	86.25	98.75	100	93.75	88.75	42.5	44.3	53.75	53.75	50.65	28.75	31.25	28.75
D.mojavensis	80	80	80	78.75	80	83.75	95	93.75	100	87.5	42.5	43.04	52.5	52.5	49.35	30	31.25	28.75
D.willistoni	77.5	77.5	78.75	78.75	78.75	86.25	88.75	88.75	87.5	100	42.5	44.3	52.5	51.25	51.95	29.63	33.33	28.4
Glossina	42.5	42.5	42.5	42.5	42.5	42.5	42.5	42.5	42.5	42.5	100	54.43	61.25	53.75	57.14	25	26.25	23.75
Musca	44.3	45.57	46.84	44.3	46.84	44.3	44.3	44.3	43.04	44.3	54.43	100	56.96	59.49	66.23	25.32	29.11	25.32
Ceratitis	48.75	48.75	48.75	47.5	48.75	53.75	53.75	53.75	52.5	52.5	61.25	56.96	100	82.5	55.84	28.75	28.75	27.5
Rhagoletis	48.75	48.75	48.75	50	48.75	52.5	53.75	53.75	52.5	51.25	53.75	59.49	82.5	100	57.14	27.5	28.75	26.25
Lucilia	49.35	50.65	51.95	50.65	51.95	46.75	50.65	50.65	49.35	51.95	57.14	66.23	55.84	57.14	100	27.27	32.47	25.97
Antheraea	28.75	28.75	30	28.75	30	27.5	28.75	28.75	30	29.63	25	25.32	28.75	27.5	27.27	100	65.43	75.31
Manduca	32.5	32.5	32.5	32.5	32.5	31.25	31.25	31.25	31.25	33.33	26.25	29.11	28.75	28.75	32.47	65.43	100	74.07
Papilio	27.5	27.5	27.5	27.5	27.5	28.75	28.75	28.75	28.75	28.4	23.75	25.32	27.5	26.25	25.97	75.31	74.07	100
Lonomia	27.5	27.5	28.75	27.5	28.75	26.25	26.25	26.25	27.5	27.16	25	25.32	27.5	23.75	27.27	67.9	60.49	62.96
Bombyx	27.5	27.5	28.75	27.5	28.75	26.25	26.25	26.25	26.25	27.16	26.25	24.05	25	25	24.68	71.6	71.6	70.37
Danaus	26.25	26.25	25	26.25	25	23.75	22.5	22.5	22.5	22.22	23.75	24.05	26.25	23.75	24.68	67.9	70.37	72.84
Apis	27.85	27.85	27.85	27.85	27.85	29.11	26.58	26.58	25.32	25	27.85	24.36	22.78	24.05	30.26	38.75	36.25	43.75
Nasonia	31.65	31.65	31.65	31.65	31.65	31.65	27.85	27.85	27.85	26.25	31.65	29.49	27.85	29.11	34.21	33.75	36.25	36.25
Tribolium	24.05	24.05	24.05	24.05	24.05	24.05	25.32	25.32	24.05	23.75	27.16	29.49	32.91	27.85	28.95	43.75	43.75	46.25
Pediculus	27.87	27.87	27.87	27.87	27.87	26.23	22.95	22.95	22.95	22.58	25	23.33	26.23	22.95	32.76	33.87	32.26	29.03
Acyrthosiphon	26.58	26.58	26.58	26.58	26.58	26.58	24.05	24.05	25.32	23.75	23.46	24.36	24.05	24.05	27.63	31.25	28.75	27.5
Collembola	21.13	21.13	21.13	21.13	21.13	19.72	19.72	19.72	19.72	19.44	21.13	22.86	23.94	23.94	23.19	22.22	19.44	19.44
Daphnia	25.33	25.33	24	25.33	22.67	21.33	16	16	16	15.79	24	22.97	24	24	23.61	19.74	18.42	17.11
Sinocyclocheilus	26.76	26.76	26.76	26.76	26.76	25.35	25.35	25.35	25.35	25	28.17	30	28.17	28.17	30.88	22.22	20.83	20.83
Carassius	26.76	26.76	26.76	26.76	26.76	25.35	25.35	25.35	25.35	25	26.76	30	28.17	28.17	30.88	22.22	22.22	19.44
Cyprinus	23.94	23.94	25.35	23.94	25.35	22.54	22.54	22.54	22.54	23.61	26.76	31.43	28.17	28.17	29.41	22.22	20.83	19.44
Labeo	27.14	27.14	28.57	27.14	28.57	25.71	25.71	25.71	25.71	26.76	25.71	31.88	28.57	28.57	34.33	22.54	19.72	18.31
Danio	23.53	23.53	25	23.53	25	22.06	23.53	23.53	22.06	24.64	23.53	30.88	25	26.47	31.82	18.84	18.84	17.39
Ciona	28.36	28.36	26.87	28.36	26.87	23.88	26.87	26.87	25.37	25	23.88	23.88	29.85	29.85	22.39	26.47	23.53	22.06
Hemotin (outgroup)	9.21	9.21	10.53	9.21	10.53	7.89	6.58	6.58	7.89	7.79	11.84	10.67	14.47	13.16	13.7	14.29	10.39	14.29

Table S2 (1/2)

	Lonomia	*Bombyx*	*Danaus*	*Apis*	*Nasonia*	*Tribolium*	*Pediculus*	*Acyrthosiphon*	*Collembola*	*Daphnia*	*Sinocyclocheilus*	*Carassius*	*Cyprinus*	*Labeo*	*Danio*	*Ciona*	*Hemotin*
Peg	27.5	27.5	26.25	27.85	31.65	24.05	27.87	26.58	21.13	25.33	26.76	26.76	23.94	27.14	23.53	28.36	9.21
D.erecta	27.5	27.5	26.25	27.85	31.65	24.05	27.87	26.58	21.13	25.33	26.76	26.76	23.94	27.14	23.53	28.36	9.21
D.simulans	28.75	28.75	25	27.85	31.65	24.05	27.87	26.58	21.13	24	26.76	26.76	25.35	28.57	25	26.87	10.53
D.yakuba	27.5	27.5	26.25	27.85	31.65	24.05	27.87	26.58	21.13	25.33	26.76	26.76	23.94	27.14	23.53	28.36	9.21
D.sechellia	28.75	28.75	25	27.85	31.65	24.05	27.87	26.58	21.13	22.67	26.76	26.76	25.35	28.57	25	26.87	10.53
D.pseudoobscura	26.25	26.25	23.75	29.11	31.65	24.05	26.23	26.58	19.72	21.33	25.35	25.35	22.54	25.71	22.06	23.88	7.89
D.grimshawi	26.25	26.25	22.5	26.58	27.85	25.32	22.95	24.05	19.72	16	25.35	25.35	22.54	25.71	23.53	26.87	6.58
D.virilis	26.25	26.25	22.5	26.58	27.85	25.32	22.95	24.05	19.72	16	25.35	25.35	22.54	25.71	23.53	26.87	6.58
D.mojavensis	27.5	26.25	22.5	25.32	27.85	24.05	22.95	25.32	19.72	16	25.35	25.35	22.54	25.71	22.06	25.37	7.89
D.willistoni	27.16	27.16	22.22	25	26.25	23.75	22.58	23.75	19.44	15.79	25	25	23.61	26.76	24.64	25	7.79
Glossina	25	26.25	23.75	27.85	31.65	27.16	25	23.46	21.13	24	28.17	26.76	26.76	25.71	23.53	23.88	11.84
Musca	25.32	24.05	24.05	24.36	29.49	29.49	23.33	24.36	22.86	22.97	30	30	31.43	31.88	30.88	23.88	10.67
Ceratitis	27.5	25	26.25	22.78	27.85	32.91	26.23	24.05	23.94	24	28.17	28.17	28.17	28.57	25	29.85	14.47
Rhagoletis	23.75	25	23.75	24.05	29.11	27.85	22.95	24.05	23.94	24	28.17	28.17	28.17	28.57	26.47	29.85	13.16
Lucilia	27.27	24.68	24.68	30.26	34.21	28.95	32.76	27.63	23.19	23.61	30.88	30.88	29.41	34.33	31.82	22.39	13.7
Antheraea	67.9	71.6	67.9	38.75	33.75	43.75	33.87	31.25	22.22	19.74	22.22	22.22	22.22	22.54	18.84	26.47	14.29
Manduca	60.49	71.6	70.37	36.25	36.25	43.75	32.26	28.75	19.44	18.42	20.83	22.22	20.83	19.72	18.84	23.53	10.39
Papilio	62.96	70.37	72.84	43.75	36.25	46.25	29.03	27.5	19.44	17.11	20.83	19.44	19.44	18.31	17.39	22.06	14.29
Lonomia	100	61.73	66.67	36.25	36.25	38.75	37.1	32.5	19.44	21.05	22.22	22.22	22.22	23.94	21.74	26.47	18.18
Bombyx	61.73	100	68.75	43.16	37.23	45.56	32.81	25.56	26.03	20.24	22.37	22.37	22.37	21.92	21.92	24.68	13.95
Danaus	66.67	68.75	100	41.05	38.3	47.78	35.94	25.56	23.29	21.43	25	25	23.68	24.66	23.29	27.27	12.79
Apis	36.25	43.16	41.05	100	53.91	45.05	34.33	24	27.4	21.18	25	26.32	23.68	24.66	27.4	24.05	13.79
Nasonia	36.25	37.23	38.3	53.91	100	41.11	34.33	25.51	24.66	24.71	27.63	27.63	23.68	26.03	26.03	27.85	12.79
Tribolium	38.75	45.56	47.78	45.05	41.11	100	31.34	26.44	24.66	22.62	28.95	28.95	28.95	28.77	26.03	30.77	13.79
Pediculus	37.1	32.81	35.94	34.33	34.33	31.34	100	34.85	30.91	28.33	23.21	23.21	23.21	25.45	20.75	28.3	10.94
Acyrthosiphon	32.5	25.56	25.56	24	25.51	26.44	34.85	100	20.55	20.73	22.37	22.37	23.68	26.03	19.18	27.03	9.76
Collembola	19.44	26.03	23.29	27.4	24.66	24.66	30.91	20.55	100	27.4	28.99	28.99	28.99	27.94	37.88	25.4	9.86
Daphnia	21.05	20.24	21.43	21.18	24.71	22.62	28.33	20.73	27.4	100	26.32	27.63	28.95	27.4	31.51	32	17.28
Sinocyclocheilus	22.22	22.37	25	25	27.63	28.95	23.21	22.37	28.99	26.32	100	93.42	89.47	87.67	58.9	28.79	6.85
Carassius	22.22	22.37	25	26.32	27.63	28.95	23.21	22.37	28.99	27.63	93.42	100	88.16	89.04	60.27	30.3	8.22
Cyprinus	22.22	22.37	23.68	23.68	23.68	28.95	23.21	23.68	28.99	28.95	89.47	88.16	100	83.56	56.16	30.3	8.22
Labeo	23.94	21.92	24.66	24.66	26.03	28.77	25.45	26.03	27.94	27.4	87.67	89.04	83.56	100	61.43	28.12	8.57
Danio	21.74	21.92	23.29	27.4	26.03	26.03	20.75	19.18	37.88	31.51	58.9	60.27	56.16	61.43	100	31.25	9.86
Ciona	26.47	24.68	27.27	24.05	27.85	30.77	28.3	27.03	25.4	32	28.79	30.3	30.3	28.12	31.25	100	12.16
Hemotin (outgroup)	18.18	13.95	12.79	13.79	12.79	13.79	10.94	9.76	9.86	17.28	6.85	8.22	8.22	8.57	9.86	12.16	100

Table S2 (2/2)

Table S2. Percent Identities by MAFFT of Pegasus peptides and the 88aa membrane peptide Hemotin, used to build the phylogenetic tree presented in Fig.S1

Peg	MKLSAVLLAIALLALSLVQCLGLPDPSTKCVMECDTQEYRSICAADDKGSTKTYRNLCVMKTENCLQNANFQKISDKECP
D.erecta	MKLSAVLLAMALLALSLVQCLGLPDPSTKCVMECDTQEYRSICAADDKGSTKTYRNLCVMKTENCLQNANFQKISDKECP
D.simulans	MKLSAVLLAMALFALSLVQCLGLPDPSTKCVMECDTQEYRSICAADDKGSTKTYRNLCVMKTENCLQNANFQKISDKECP
D.yakuba	MKLSAVLLAIALLALSLVQCFGLPDPSTKCVMECDTQEYRSICAADDKGSTKTYRNLCVMKTENCLQNANFQKISDKECP
D.sechellia	MKLSAVLLAMALFALSLVQCLGLPDPSTKCVMECDTQEYLSICAADDKGSTKTYRNLCVMKTENCLQNANFQKISDKECP
D.pseudoobscura	MKLFAVFLALALYALTVVQGLSLPDPNVKCAMDCDTQEYAAICAADDKGTTRTYRNLCVMKTENCLQNASFQKISDKECP
D.grimshawi	MKLFAVFLALTLYALTLVQCLSLPDPNVKCAMECDTQENKYICAADDKGTTKTYRNVCIMKTENCLQNANFQKISDKECP
D.virilis	MKLFAVFLALTLYALTLVQCLSLPDPNVKCAMECDTQESKYICAADDKGTTKTYRNVCIMKTENCLQNANFQKISDKECP
D.mojavensis	MKLFAVFLALTLYALTLVQCLSLPDPNVKCNMECDTQENQFICAADDKGVTKTYRNVCIMKTENCLQNANFQKISDKECP
D.willistoni	MKLFAVFLTLALFALTTLVQGFSLPDPNVKCSMECDTQEDAAICAADDKGTTKTYRNVCIMKTENCLQNANFQKISDKECP
Glossina	MNFISVCIKVAMIVLALALVVYSSPLTDNKRNCQMNCELQDYKPICGTDDAGVTRTFNNICILKTENCLRTYTFQKTGDGVCP
Musca	MKLFPLFLAMAIFVISFVNSAPAPGDTECEFNCDLQDYKPVCGTDDSGDTKTFNNMCIIKTENCLRKTSYQKTGDGDCP
Ceratitis	MKFFAIIITLALYAFALVHSLAISDTNVKCQMNCDLQDYKPVCGTDDAGETKSFNNLCILKTENCLRQLSFQKTADGECP
Rhagoletis	MKLYAVLLAFVLYAITFVHSFAISDTNVKCQLNCDLQDFKPVCGTDDAGETKSFNNLCILKTENCLRQLSFQKTADGECP
Lucilia	MKLLALFLAMAIFILPLISAFPKGRDCEVHNCELQDYKPICGTDDKGDTKTFTNYCILKTENCLRNQNYQKTADGECP
Antheraea	MKATTLIIFAVLFVASDLKQVKAEAARKKACLHDCTNIKFQPICASKDGDKPKSFGSVCVMNNYNCEHKDTLRKISDGECA
Manduca	MKTVALFLFAALIVATDLKQLKADKARKNACLHDCSGVDFDPICAGKQGEKPKSFGNECVMNNYNCENKDNLHKISKGQCP
Papilio	MKYFTLFIFAVLVVSQDLKQLKAEAARKKACTQDCSSAKFEPICAGKQGEKPKSFGSECVMNNYNCEHKDTLHKISKGECP
Lonomia	MKPATLIIFAVLFVAVNLTNLKAQAARQRACLSDCSTVPYQPICAGKTGDKPKSFANVCVMNNHNCEHRDTLAKISNGACS
Bombyx	MKTIIFCAFAVLFVAVSCRPDKLDLKQVKADAERKKACMHDCTKANLDPICAGKTGEKPKSFGNECVMNNYNCEHKDTLRKISQGQCPGSDGIRLS
Danaus	MKAATLIVFAVMLVAVSCRPDKPDLKQLKAEAARKKACLQDCSSVKYDPVCAGKPGEKSKSFGNECVMNNHNCENNDTLHKLSKGQCAGSDSIRLS
Apis	MKSIVLLALVVACALVLAETASTSSSTKPVKEPKEIKEAATASKDVKAAKEAAKKKKDCARECGTAYEPICAHDPNDATSKPRTFGTECALNVHNCEMGTKLVMKSKGECPGSGGVRLS
Nasonia	MKLAFALGAVLLVCLVCSIEAGPTEKPVTTAKPTASKEVKDAIKNAKEAKKAKKDCLKSCPTDYVPICAHDPANASFKPRTFGNQCVLDTHNCEMGTKLVMKMKGECPGSDGVRL
Tribolium	MKFAVCFVVLVAVVASSFALPAKGSTKAALKKKPCLKDCGDVYKPVCAGDGSAKNNKSFGSECVLSNYNCETGNNLKIQSQGECPGGGGVRLS
Pediculus	MVAIASVHGLPGGGKNKLKGQQQQQCPNICPANYDPVCAADLNDSKSSPRLFGNKCVLNTHNCEHNSRKY
Acyrthosiphon	MKSLTLVLLVGLTVALVSVNGSPAGDKKIGTSHKMKTTSTTTTLSPARKCKSECPNDYNPICGSDGAKVNLSFGNKCVMEKYNCEHNASEYRGQSVDDINGL
Collembola	MVRNLFLGFLVVALVAMIATGEDSQNDRKPAGRAICTKEYSPVCGSDGKTYGNKCEFNNALQLIKDLTFKEGPCQ
Daphnia	MGQLISMKFVFSVMFFVFLGTCILTGQSGVNGVLIGSRRCGIICTREYRPVCGTDGRTYPNLCVLRAKNLCYGTRIRKAYKDSNSSIDVEI
Sinocyclocheilus	MKLAILICVSVLVYLSVAEAQQSEDNTVPDFGCTREYNPVCGDDGVTYSNECMLHWENKSRDKNVSLKHVGVCETS
Carassius	MKLAILICVSVLIYLSVAEAQQSEDNNVPDFGCTREYNPVCGDDGVTYSNECMLHWENKIRGKNVSLKHVGKCETS
Cyprinus	MKFAMLVCVSVLFYISVAETQQSEDNNVPDFGCTREYNPVCGDDGVTYSNECMLHWENKHRDKNVSLKHVGVCETS
Labeo	MKLAILICVSVLFYLSVAEAQESENNVPEFGCTREYNPVCGDDGLTYSNECMLHWENKVRNKNVSLKHAGRCE
Danio	MKLAILVCVSVVFYLTMVEAEATDESPIVCTREYKPVCGDDGKTYSNECMLRWETNAKEVVVNVKHEGKCESS
Ciona	MKVTFALVLVVLLAASSVDALPKCFIPCPRHIKPVCGSDGSNLVWYANECGMRVAACHLGSAITKVNEATCSSVKNPFNYRS
Hemotin (outgroup)	MDCFKVFEVVFQSEINPLLLIPAVATIALTLCCYCYHGYQWIRDRRTARIEEQQAQLPLPLSRISITPGCSMVATTKLTHSRNSVDIY

Table S3

Table S3. Amino acid sequence of Pegasus peptides from the species analysed in table S2, identified in our homology search.

Used for:		Name:	Sequence:
pegGFP construct	cDNA PCR for TOPO cloning	CG17278 Fw	5' CACCAATTGACAGCGGTCAGCGAT 3'
		CG17278 Rv	5' TTCGGACACTCCTTGTCGCT 3'
Danio Peg-GFP construct	cDNA PCR cloning	zPeg Dr Fw	5' CACCTGCTGAGTTTCCCACT 3'
		zPeg Dr Rv 1	5' CTGCAGCATTTTGGCACACT 3'
	NEB HI-FI assembly	5´UTR_Fw	5' CTCTCTTCTCTTCTCTCTCTCTTTCTCGAGGTCATCAAGCTTAG 3'
		5´UTR_Rv	5' TGGCGAGCTTCATGTTGTTACTAGTTGGTTTAGTTTTC 3'
		GFP_Fw	5' CGAGAGCTCTAAAAGCTCCGCCACCATGGTG 3'
		GFP_Rv	5'TTTAGGTATAATGTTATCAAGCTCCTCGAGTTAACGTTACGTTAACGTTAACGTTCGAG 3'
		Danio CDS_Fw	5' ACTAGTAACAACATGAAGCTCGCCATCCTG 3'
		Danio CDS_Rv	5' GGTGGCGGAGCTTTTAGAGCTCTCGCATTTTCC 3'
Peg CRISPR mutant	Guide RNA	chi-CG17278_ORF_guide_1_Fw	5' CTTCGTACCGATCCATTTGCGCTG 3'
		chi-CG17278_ORF_guide_1_Rv	5' AAACCAGCGCAAATGGATCGGTAC 3'
Peg-Gal4	Tandem RNA guides	Tan-CG17278_3'guide_Rv	5'TATATAGGAAAGATATCCGGGTGAACTTCGGAAATAACTTAATCATGGAGTTTTAGAGCTAGAAATAGCAAG 3'
		Tan-CG17278_5'guide_Fw	5'ATTTTAACTTGCTATTTCTAGCTCTAAAACCTTCATCTGGTGTATTCCGGCGACGTTAAATTGAAAATAGGTC 3'
	Intron amplification	intron_fragment1_Fw	5' CTAGTAACAACTAGAAGCTCTCAGCAGTATTG 3'
		intron_fragment1_Rv	5' TGCGGCCGCTCCGGAGAATTCATGATTAAGTTATTTCCGCTG 3'
Q-RT-PCR		Wg_Fw	5' CCAAGTCGAGGGCAAACAGAA 3'
		Wg_Rv	5' TGGATCGCTGGGTCCATGTA 3'
		rp49_Fw	5' AGCATACAGGCCCAAGATCG 3'
		rp49_Rv	5' TGTTGTCGATACCCTTGGGC 3'

Table S4

Table S4. List of primers used in this manuscript.

Supplementary references:

1 Magny, E. G. *et al.* Conserved regulation of cardiac calcium uptake by peptides encoded in small open reading frames. *Science* **341**, 1116-1120 (2013).

2 Pueyo, J. I. *et al.* Hemotin, a regulator of phagocytosis encoded by a small ORF and conserved across metazoans. *PloS Biology* **14**, e1002395, doi:10.1371/journal.pbio.1002395 (2016).

3 Gonzalez, F., Swales, L., Bejsovec, A., Skaer, H. & Martinez Arias, A. Secretion and movement of wingless protein in the epidermis of the Drosophila embryo. *Mech Dev* **35**, 43-54 (1991).

4 Couso, J. P., Bishop, S. A. & Martinez Arias, A. The wingless signalling pathway and the patterning of the wing margin in Drosophila. *Development* **120**, 621-636 (1994).

5 Couso, J. P., Bate, M. & Martinez Arias, A. A wingless-dependent polar coordinate system in Drosophila imaginal discs. *Science* **259**, 484-489 (1993).

6 Couso, J. P., Knust, E. & Martinez Arias, A. Serrate and wingless cooperate to induce vestigial gene expression and wing formation in Drosophila. *Curr Biol* **5**, 1437-1448 (1995).

7 Diaz-Benjumea, F. J. & Cohen, S. M. Interaction between dorsal and ventral cells in the imaginal disc directs wing development in Drosophila. *Cell* **75**, 741-752 (1993).

8 Klein, T. & Martinez Arias, A. The vestigial gene product provides a molecular context for the interpretation of signals during the development of the wing in Drosophila. *Development* **126**, 913-925 (1999).

9 Neumann, C. J. & Cohen, S. M. Distinct mitogenic and cell fate specification functions of wingless in different regions of the wing. *Development* **122**, 1781-1789 (1996).

10 Zecca, M. & Struhl, G. Recruitment of cells into the Drosophila wing primordium by a feed-forward circuit of vestigial autoregulation. *Development* **134**, 3001-3010, doi:10.1242/dev.006411 (2007).

11 Piddini, E. & Vincent, J. P. Interpretation of the wingless gradient requires signaling-induced self-inhibition. *Cell* **136**, 296-307 (2009).

12 Phillips, R. G. & Whittle, J. R. wingless expression mediates determination of peripheral nervous system elements in late stages of Drosophila wing disc development. *Development* **118**, 427-438 (1993).

13 Alexandre, C., Baena-Lopez, A. & Vincent, J. P. Patterning and growth control by membrane-tethered Wingless. *Nature* **505**, 180-185 (2014).
