# Supplementary material for: Pegasus, a small extracellular peptide enhancing short-range diffusion of Wingless
Source: Nat Commun. 2021 Sep 27;12:5660. doi: 10.1038/s41467-021-25785-z (PMC8476528; doi:10.1038/s41467-021-25785-z)
Supplement: Supplementary file 3 — Description of Additional Supplementary Files [file 41467_2021_25785_MOESM3_ESM.pdf]

Description of Additional Supplementary Files

File Name: Supplementary Movie 1
Description: Live imaging of WgGFP diffusion, activated by ptc-GAL4 UAS-Gal80ts, 24h before dissection, in a wt background, immediately after photobleaching.

File Name: Supplementary Movie 2
Description: Live imaging of WgGFP diffusion activated by ptc-GAL4 UAS-Gal80ts, 24h before dissection, in a peg null background, immediately after photobleaching.
