# Supplementary material for: Pegasus, a small extracellular peptide enhancing short-range diffusion of Wingless
Source: Nat Commun. 2021 Sep 27;12:5660. doi: 10.1038/s41467-021-25785-z (PMC8476528; doi:10.1038/s41467-021-25785-z)
Supplement: Supplementary file 6 — Reporting Summary [file 41467_2021_25785_MOESM6_ESM.pdf]

nature research

Corresponding author(s): Juan Pablo Couso

Last updated by author(s): 15/06/2021

Reporting Summary

Nature Research wishes to improve the reproducibility of the work that we publish. This form provides structure for consistency and transparency in reporting. For further information on Nature Research policies, see our Editorial Policies and the Editorial Policy Checklist.

Statistics

For all statistical analyses, confirm that the following items are present in the figure legend, table legend, main text, or Methods section.

n/a	Confirmed	
☐	☒	The exact sample size (n) for each experimental group/condition, given as a discrete number and unit of measurement
☐	☒	A statement on whether measurements were taken from distinct samples or whether the same sample was measured repeatedly
☐	☒	The statistical test(s) used AND whether they are one- or two-sided<br>*Only common tests should be described solely by name; describe more complex techniques in the Methods section.*
☒	☐	A description of all covariates tested
☒	☐	A description of any assumptions or corrections, such as tests of normality and adjustment for multiple comparisons
☐	☒	A full description of the statistical parameters including central tendency (e.g. means) or other basic estimates (e.g. regression coefficient) AND variation (e.g. standard deviation) or associated estimates of uncertainty (e.g. confidence intervals)
☐	☒	For null hypothesis testing, the test statistic (e.g. F, t, r) with confidence intervals, effect sizes, degrees of freedom and P value noted<br>*Give P values as exact values whenever suitable.*
☒	☐	For Bayesian analysis, information on the choice of priors and Markov chain Monte Carlo settings
☒	☐	For hierarchical and complex designs, identification of the appropriate level for tests and full reporting of outcomes
☒	☐	Estimates of effect sizes (e.g. Cohen's d, Pearson's r), indicating how they were calculated

Our web collection on statistics for biologists contains articles on many of the points above.

Software and code

Policy information about availability of computer code

Data collection	No custom computer software or code was used to collect data for this study
Data analysis	Wolfram Mathematica (v 10.4) was used to obtain the reaction diffusion model for Wg effective transport. Otherwise all graphs, and data analysis were obtained with Graphpad Prism (v5.00). ImageJ (v1.8.0_66) was used for image analysis.

For manuscripts utilizing custom algorithms or software that are central to the research but not yet described in published literature, software must be made available to editors and reviewers. We strongly encourage code deposition in a community repository (e.g. GitHub). See the Nature Research guidelines for submitting code & software for further information.

Data

Policy information about availability of data

All manuscripts must include a data availability statement. This statement should provide the following information, where applicable:

- Accession codes, unique identifiers, or web links for publicly available datasets
- A list of figures that have associated raw data
- A description of any restrictions on data availability

All relevant data are available from the authors

Field-specific reporting

Life sciences study design

All studies must disclose on these points even when the disclosure is negative.

Sample size	For brsitle quantifications, at least 20 wings of different animals were considered per genotype. And for nuclear armadillo signal quantification, at least 15 cells were quantified per field of observation, per genotype. Each expression profile plot represents the mean of at least 5 different imaginal discs from different animals. These numbers were enough to obtain significant differences as evaluated by standard one tailed t test, with acceptable standard deviations.
Data exclusions	No data was excluded.
Replication	The reported data was reproducible in all samples tested. Representative micrographs from unquantified data (antibody stainings and in-situ hybridisation) presented in this manuscript were used when similar results could be observed in all imaginal discs examined, in two independent experiments.
Randomization	Randomization was not relevant to this study. All experiments were performed on individuals of specific genotypes, and data was collected using identical parameters and quantification methods.
Blinding	Blinding was not possible as all observations were made in animals of specific genotypes.

Reporting for specific materials, systems and methods

We require information from authors about some types of materials, experimental systems and methods used in many studies. Here, indicate whether each material, system or method listed is relevant to your study. If you are not sure if a list item applies to your research, read the appropriate section before selecting a response.

Materials & experimental systems

n/a	Involved in the study	
☐	☒	Antibodies
☒	☐	Eukaryotic cell lines
☒	☐	Palaeontology and archaeology
☐	☒	Animals and other organisms
☒	☐	Human research participants
☒	☐	Clinical data
☒	☐	Dual use research of concern

Methods

n/a	Involved in the study	
☒	☐	ChIP-seq
☒	☐	Flow cytometry
☒	☐	MRI-based neuroimaging

Antibodies

Antibodies used	Anti-Wg 4D4 (mouse, DSHB: 4D4-S, used at 1:50) and anti-Arm 7A1 (mouse, DSHB: 7A1-S used 1:50) were obtained from The Developmental Studies Hybridoma Bank, at the University of Iowa (https://dshb.biology.uiowa.edu/). Anti-Sens (Guinea-Pig, used 1:3000) was a gift from Takashi Koyama. We used the following secondary antibodies: to detect Wg we used anti-mouse biotin, (Jackson (, 715-065-151, 1:200), and avidin-Cy5 or avidin-FITC, (Jackson, (016-220-084, 1:1000) or (avidin-Alexa 488 (Jackson, 016-540-084, 1:500) respectively,), for other antigens we used anti mouse-Rhodamine, (Jackson (, 715-025-150, 1:250), and anti guinea-pig-rhodamine, (Jackson, (106-025-003, 1:250).For nuclear labelling we used DAPI at a final concentration of 300 nM. For western blots and imuno-precipitations we used rabbit anti-GFP 1:2500 (Invitrogen, MA5-15256, 1:2500), mouse anti-GFP 1:2500 (Roche, 11814460001, 1:2500), and anti-HA 1:5000 (Roche, 12CA5 ,1:5000). For in situ hybridization, we used anti-mouse-HRP (Donkey, Jackson, 715-035-150, 1:10000).
Validation	Anti-sens was validated in (Cell 102, 349-362 (2000)). Anti Arm was validated in (Cell 63.3 (1990 Nov 2): 549-60.). Anti Wg was validated in (Science (New York, N.Y.) 273.5280 (1996 Sep 6): 1373-7). All commercial antibodies used in this manuscript have been thouroughly validated, and used for similar apllications as those in this manuscript in several peer reviewed articles.

Animals and other organisms

Policy information about studies involving animals; ARRIVE guidelines recommended for reporting animal research

Laboratory animals	Fly stocks and crosses were cultured at 25ºC, unless otherwise stated. For time-specific activation of gene expression with the Gal80ts system, the cultures were carried out at 18ºC and then shifted to 29ºC prior to dissection after the indicated times. The following lines were obtained from the Drosophila Bloomington stock centre at Indiana University (https://bdsc.indiana.edu/): Or-R (used as wt), w;;69BGal4 (#1774), w;;UASdsRed (#6282) , w;enGal4,UAS-dsRed /CyO (#30557), w hsFlp;; FRT 82B tubGFP (#5188), da-Gal4 (#55851), w;UAS-mCD8-FRP(#86558) and w; NotumI6/ TM3 (#4117). The UAS-sgl stock (M{UAS-sgl.ORF.3xHA}ZH-86Fb, #F003098) was obtained from flyORF (https://flyorf.ch/). The w;ptcGal4-tubGal80ts ;MKRS/TM6b was a gift from James Castelli Gair-Hombria, the w; UAS-wgGFP and w; wgNRT w; dlpMH20/TM6b, and UAS dlp-HA stocks were gifts from Jean Paul Vincent. rn-Gal413 was previously published in St Pierre et al. 200239. wgIL homozygous flies raised at 17oC were obtained as in Couso et al. 1994.
Wild animals	This study did not involve wild animals

Field-collected samples	This study did not involve field samples
Ethics oversight	No ethics oversight was needed for this study.

Note that full information on the approval of the study protocol must also be provided in the manuscript.
